# Mate Recognition and Expression of Affective State in Croop Calls of Northern Bald Ibis (*Geronticus eremita*)

**DOI:** 10.1371/journal.pone.0088265

**Published:** 2014-02-05

**Authors:** Georgine Szipl, Markus Boeckle, Sinja A. B. Werner, Kurt Kotrschal

**Affiliations:** 1 Department of Cognitive Biology, University of Vienna, Vienna, Austria; 2 Core Facility Konrad Lorenz Forschungsstelle for Behaviour and Cognition, University of Vienna, Grünau, Austria; 3 Department of Behavioural Biology, University of Vienna, Vienna, Austria; Universite Paris Sud, France

## Abstract

Northern Bald Ibis are socially monogamous and year-round colonial birds with a moderate repertoire of calls. Their ‘croop’, for example, is used during greeting of mates, but also during agonistic encounters, and provides an ideal case to study whether calls are revealing with respect to motivational states. We recorded croop calls in a semi-tame and free-roaming flock of Northern Bald Ibis in Austria, and analysed the vocal structure to identify parameters (e.g. call duration, fundamental frequency) potentially differing between social contexts, sexes and individuals. Additionally, we conducted playback experiments to test whether mated pairs would discriminate each other by their greeting croops. Acoustic features showed highly variable temporal and structural parameters. Almost all calls could be classified correctly and assigned to the different social contexts and sexes. Classification results of greeting croops were less clear for individuality. However, incubating individuals looked up more often and longer in response to playbacks of the greeting calls of their mate than to other colony members, indicating mate recognition. We show that acoustic parameters of agonistic and greeting croops contain features that may indicate the expression of affective states, and that greeting croops encode individual differences that are sufficient for individual recognition.

## Introduction

Individual recognition is important in many social contexts [1], and several sensory channels can be used for this purpose, either separately (visual: e.g. [2], and olfactory: [3], [4]) or combined (visual and acoustic: [5]). In birds, the auditory domain has been studied extensively. However, most studies on acoustic individual recognition have been conducted in oscine passerines, which have large and complex song and call repertoires. In non-passerines, which have received comparatively less attention in acoustic studies, individually distinct vocalisations may rely on morphometric differences with a high genetic influence [6]. Acoustic individual recognition in non-passerines was shown in penguins [7]–[9], other seabirds [10], and suboscines [11], revealing highly complex vocal systems also in non-passerines. Indeed, individual recognition is not limited to the phylogenetic taxonomy but should rather evolve whenever social context requires repeated individualised interactions [1]. In socially monogamous birds, biparental care is common [12] and requires repeated interactions among mated pairs, as both have to coordinate actions to optimise their investment [13],[14]. Especially in group-living birds, which often breed in dense colonies with many other conspecifics, discriminating the mate from others is a challenging task. Therefore the ability of individual recognition should be beneficial and selected for [15].

Aside from individuality, which requires stable individual differences, acoustic signals can be modified by several external factors: seasonal variation [16], but also group size and composition [17], [18] can cause variations in vocalisations. Lately, acoustic studies showed that internal factors like the physical constitution [19], or the emotional/motivational state can influence vocal signals. Motivational state was suggested to alter the structure of mammalian and bird vocalisations in different contexts almost forty years ago [20]. However, the basic element of sender motivation is supposed to be the underlying emotional state, being an integral element of motivation [21]. A recent framework for studying animal emotions suggests to consider both arousal level and positive and negative valence, which vary along two different dimensions, as well as behavioural, cognitive, and neurophysiological components, when studying animal emotions [21]. While differences between motivational call types could encode valence, differences within one call type would rather indicate different levels of arousal [22]. It was shown in humans and mammals, that physiological arousal manifests mainly in varying temporal parameters of calls (e.g. duration, call rate), and in features related to fundamental frequency (reviewed in [23]). The latter was shown to be encoded not only in human speech, but also in other mammal vocalisations (primates: [24],[25], humans: [26], Tree Shrews, *Tupaia belangeri*: [27], African elephants, *Loxodonta africana*: [28]). Those studies all revealed, among other parameters, elevated measures in fundamental frequency with rising aversion, indicating the existence of measurable overall characteristics of arousal in communication signals between mammal taxa. In contrast, emotional valence, which is rarely studied, may be reflected in differences in intonation and energy distribution within vocalisations (reviewed in [23]).

Northern Bald Ibis (*Geronticus eremita*) are socially monogamous and year-round colonial birds [29]. They forage in flocks and usually breed in dense colonies [30]. During breeding season, individuals defend their nest against conspecifics, as aggression between breeding pairs was reported as a significant cause for egg loss and nest destruction [31]. Almost extinct in the wild, the Northern Bald Ibis is listed on the global IUCN red list as critically endangered species [32]. Currently, one colony of approximately 100 breeding pairs still exists in the wild in Morocco [30], and a relict colony comprising two breeding pairs has been discovered in Syria [33]. The acoustic repertoire of Northern Bald Ibis features three main call types, which occur in several social contexts. Calls do not seem to be discrete, as gradual conversions between call types have been described [34]. One of these call types, the ‘croop’ or ‘chrup’, is used during courtship and when greeting mates as well as during agonistic interactions of colony members over food, mates or nesting sites [34], [35], [36]. Greeting is a ritualised display in which mates alternate in uttering croops, bow their heads and occasionally offer nesting material to their mate or engage in mutual preening [37]. Pegoraro & Föger [36] investigated croops in an aviary-kept zoo population and showed that agonistic croops were significantly longer than greeting croops and varied in several frequency measurements, suggesting that agonistic and greeting croops should be treated as two classes of calls. They found sex and individual differences in the greeting calls, and further suggested that croop calls may transmit various messages about the motivational state of the signaller [36]. Hence, Northern Bald Ibis provide an promising model system to study two calls that sound very similar but are modified by different social contexts, and might contain stable features for individual recognition. However, in the former study [36], croop parameters were measured manually from printed spectrograms. For that, calls were sectioned into three frequency ranges between 0 to 8000 Hz, and frequency measures were given for each of the three sections. This approach comes with a wide margin of possible errors and makes replication for comparative studies difficult. Additionally, it has not been tested so far whether Northern Bald Ibis can actually perceive individual differences in these calls.

In our present study, we recorded croops in a semi-wild and free-roaming flock of Northern Bald Ibis in both agonistic and greeting contexts and performed a detailed analysis on the source- and filter-related vocal structure to see which parameters differed between croops uttered in different social contexts and by different sexes and individuals. We further used greeting croops in a paired playback study two years later to test whether birds could discriminate between calls of colony members and recognise the calls of their mates.

## Materials and Methods

### Ethics Statement

No permits were required for the described study, which complied with all relevant regulations. Birds are used to human presence inside the aviary and were not disturbed in their breeding activity.

### General Information

In 2008, the semi-wild, free-flying and non-migratory colony of Northern Bald Ibis at the Konrad Lorenz Forschungsstelle (KLF; 47° 48′ N, 13° 56′ E) in Gruenau, Austria, consisted of 35 individuals. The group was established for research purpose in 1997 by hand-raising zoo-bred hatchlings [38]. A large outdoor aviary, situated at the Cumberland Gamepark approximately 1 km from the KLF serves as breeding and roosting place. Birds are free to enter and leave the aviary, but may be locked in a few weeks in snowy winters for protection against aerial predators. At present, the colony reproduces independently and the population is steadily increasing, with 46 individuals after the breeding season in 2012. The colony is provided supplemental food during winter from late October to the end of March. Breeding usually begins in mid-March and both males, and females share parenting duties [37]. All birds can be discriminated individually by coloured leg bands.

### Call Recordings

Calls were recorded in March 2008, shortly before breeding season, with a Sennheiser directional microphone (ME67 long gun microphone on a K6 module) and a handheld digital recorder (Marantz PMD660) on meadows adjacent to the KLF, where the birds often forage. The recordings were conducted by SABW from close distance (one to two meters) with the microphone directed towards the birds. The identity of the caller and the social context were spoken into the microphone. Social contexts were easily distinguishable as agonistic croop calls are accompanied by head bowing, bill shaking, and threatening/pecking towards the opponent, whereas greeting croops are part of extensive ritualised displays including mutual preening and offering of nesting material [37]. Digital audio files were recorded with a sampling rate of 48 kHz and a 16-bit dynamic range.

### Playback Experiment

Playbacks were conducted between 0700 in the morning and 1800 in the evening within the aviary at the Cumberland game park in May 2010 during breeding season, using the greeting croop calls recorded in 2008. Out of the 15 established breeding pairs in 2010, we used 12 mated and incubating birds as focal individuals (4 males, 8 females) of which greeting calls of their mates were available from the recordings two years before. Due to the limited number of calls available, we tested 3 entire pairs (6 individuals) and 6 mated individuals, where only one bird of the pair was tested. We designed a paired playback experiment in which each focal bird received, depending on the number of calls available, 2 to 6 playback sessions consisting of two calls; one greeting croop of the mate and one of a non-mate colony member (mean number of sessions +SD = 4.92+1.68, total number of sessions  = 59). The silence interval between two calls in one session was five minutes. In each session, we used different calls of the same mate and calls of different non-mates as stimuli. Stimuli were counterbalanced between individuals and sessions. For the playbacks, we selected calls with a good signal-to-noise ratio, which were processed with an 80 Hz high-pass filter to diminish background noise. The files used for playback were uncompressed. wav files (sampling rate  = 48 kHz, dynamic range  = 16-bit) created with Adobe Soundbooth CS4 for Mac. We used an iPod nano (Model No: A1320) and a K82 active speaker (dB Technologies; frequency response  = 90 Hz – 18 kHz), which was placed inside the aviary at 3–4 meter distance and out of sight of the birds. Stimuli were played back with sound pressure levels of approx. 60 dB measured at 1 meter (Voltcraft SL-100) while the focal bird was incubating and its mate and the non-mate colony member had left the aviary for foraging. As mated pairs share incubation, one partner is incubating while its mate leaves the nest site (in this case the aviary) to forage on meadows several kilometres away. Thus by checking that stimuli birds were not present we assured that these birds were out of acoustic and visual range of the focal individual before each session. Minimal interval between sessions was 10 minutes. We videotaped behavioural responses of the focal birds during and after the playback (e.g. look up, turn head) for later coding.

### Data Analyses

After inspecting the call structure in the spectrograms, we decided on extracting source- and filter-related acoustic parameters using a custom built routine in Praat (version 5.1.25 [39]). We measured minimum, maximum, mean values, and range of the fundamental frequency as well as harmonics-to-noise ratio (HNR; a relation of energy in harmonics to energy in noise), jitter (a measurement of random variations of periodicity of the acoustic source), call duration, and duration of tonal parts. Intensity-related call features were transformed into relative amplitude changes and we extracted relative amplitude range and changes over time within the calls. To increase precision in comparison to previous measurements [36], where three frequency ranges accumulating several frequency bands, we conducted a detailed formant analysis. Formants are resonances produced in the vocal tract. The unextended vocal tract length of Northern Bald Ibis is approximately 12 cm (measured from dissected vocal tracts; N = 8, Boeckle et al. unpublished data). From this, resonant frequencies can be estimated with a formula that uses a uniform tube with one end closed [40]: the first formant equals the speed of sound in air (350 ms^−1^) divided by four times the length of the vocal tract; higher formants are odd integer multiples of the first formant. We measured mean frequencies of the first four formants below 6500 Hz. From this we calculated mean formant dispersion (the spacing of formant frequencies).

We used a Fast Fourier transform (FFT) method with a Gaussian window shape (dynamic range  = 70 dB, number of time steps  = 1000, number of frequency steps  = 250, window length  = 0.015 s) for all spectrographic representations of the calls. The measured variables were automatically logged into an output file. For a detailed description on the commands used in Praat, see File S1.

Videos of the playback experiment were blind-coded by GS using Solomon Coder beta version 13.09.09 (Copyright by András Péter; http://solomoncoder.com), measuring the occurrence of a response (yes/no) and response duration (up to 10 seconds). Response was defined as the focal bird stopping former action (e.g. resting, preening) and looking up or into the direction of the speaker.

### Statistics

We used nonparametric statistics to identify important parameters, as most measured parameters were not normally distributed. The influences of call context (greeting or agonistic) on call characteristics were analysed using a Wilcoxon singed rank test. Sex and individuality (separately for males and females) were investigated using Mann-Whitney U and Kruskal-Wallis tests. Discriminant Function Analyses (DFA) were conducted by applying the leave-one-out cross validation method. For DFA, we used the hold-out-sample method and randomly chose 75% of the calls of each individual (termed selected cases) to calculate discriminant functions. The remaining 25% of the calls were then used for classification tests (termed unselected cases). Both selected and unselected cases were equally distributed among individuals, sexes and contexts to meet the 75% and 25% criterion. To account for different distributions of calls among categories (social context, sex, individuals), we weighted cases for conducting DFAs by calculating the proportion of calls per category. Prior probabilities were calculated from group size. Coefficients of parameters lower than the maximum F value of 3.84 were removed from the model; the minimum F value was 2.71. The step-wise method was applied with parameters that showed highest significant variability in the previous tests. A total of 146 croop calls of 16 males and 5 females were analysed for DFA. In the greeting context, 112 calls were recorded, with a mean of 6 calls per male and 7 calls per female. During agonistic interactions, 34 calls were uttered, with a mean of 3.6 calls per male and only one call of one female. Due to low sample size of agonistic croop calls in females (*N* = 1), a DFA was calculated for calls in different social contexts only in males (*N_greeting_*  = 84, *N_agonistic_*  = 33). To test for differences in sex and between individuals, we used greeting croop calls. In the DFA testing classification of sexes, 112 greeting calls were used (*N_male_*  = 84, *N_female_*  = 28). To examine inter-individual differences within males and females two DFA were calculated for both sexes separately. In males, we used 82 calls of 12 birds (two individuals with low numbers of greeting calls were excluded from analysis). In females, 28 calls of 4 individuals were used. Chi^2^ tests were used to calculate differences between correct classifications and prior probabilities. In addition to DFA, permutated DFA [pDFA; 41] with 1000 permutations and 100 random selections were conducted for social context and sex. The same parameters used in DFA were entered in pDFA, but here we controlled for individual identity when testing for differences in social context and sex. For social context, a crossed pDFA was calculated on a reduced set of calls (*N* = 52) of 5 individuals of which calls in both contexts were available. For sex, a nested pDFA was used on the same set of calls as for the DFA examining sex discrimination.

To analyse differences in responses and response duration onto the playback stimuli, we calculated two generalised linear mixed models (GLMMs). For the analysis of response (yes/no), a binomial error distribution with a logit link function was chosen. Response duration was analysed with a Gamma distribution and a log link function using all cases where responses occurred. Behavioural response and response duration of the focal bird were used as target variables. To account for repeated measures and the different occurrences of mate and non-mate stimuli in the playback study design, individual identity of focal and stimuli birds were included as random factors. Session (1–6), sequence (the order of stimuli within a session, i.e. mate or non-mate played back first) and the status of the stimulus bird (mate vs. non-mate) were entered as fixed factors. We used a backward step-wise procedure, starting with the full model including all fixed factors and all two-way interactions between them. Non-significant factors and interactions were excluded step by step. The final model was determined by the lowest second order Akaike's information criterion (AICc) value. All factors that remained in the final model are presented in the results section. Statistical analyses were performed in SPSS 19.0 and R 3.0.1 [42].

## Results

### Social Context

**Figure 1:**
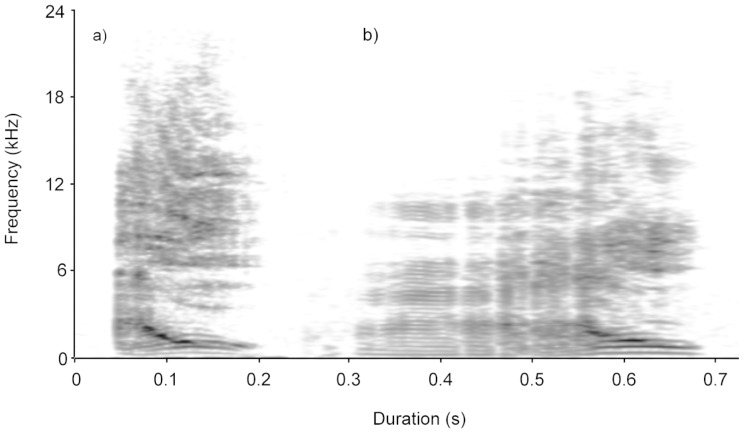
Example of one spectrogram of an agonistic (a) and a greeting (b) croop call. Spectrogram settings: FFT method, Gaussian window shape, window length  = 0.015 s, time steps  = 1000, frequency steps  = 250, dynamic range  = 70 dB.

Greeting croops were significantly longer (Wilcoxon singed rank test: *Z* = −2.366; *P* = 0.018), had lower mean fundamental frequencies (*Z* = −2.197; *P* = 0.028), but revealed higher frequencies for the second formant (*Z* = −2.366; *P* = 0.018) than croops emitted during agonistic encounters. Agonistic croops showed greater relative amplitude ranges (*Z* = −2.197; *P* = 0.028), and more rapid relative amplitude changes over time (*Z* = −2.366; *P* = 0.018) than greeting croops (Table 1). For a spectrographic representation of both call types, see Figure 1. Based on mean fundamental frequency, second formant, call duration, and relative amplitude changes over time, 95.3% of the unselected greeting and agonistic croops were classified correctly by DFA (Table 2a). Compared to the classification expected from prior probabilities (greeting calls  = 66.6%, agonistic calls  = 33.4%) the level of classification was statistically significant (χ^2^ = 17.0315; df = 1; *P*<0.001). In the crossed pDFA on the reduced set of 52 calls of 5 individuals, 35.33 of the cross-validated calls (67.94%) were classified correctly (*P* = 0.039), using the same parameters (mean fundamental frequency, second formant, call duration, and relative amplitude changes over time).

**Table 1:** Mean values and standard deviation of measured parameters describing croop calls in different social contexts (*N* =  number of calls).

Parameters	Social context			
	Greeting (*N* = 112)		Agonistic (*N* = 34)	
	Mean	SD	Mean	SD
Mean fundamental frequency (Hz)	264.03	29.89	308.22	31.25
Maximum fundamental frequency (Hz)	315.59	49.07	359.74	41.66
Minimum fundamental frequency (Hz)	221.08	14.64	240.92	23.35
Fundamental frequency range (Hz)	94.51	42.53	118.82	35.22
Formant 1 (Hz)	970.81	206.23	1051.16	221.21
Formant 2 (Hz)	2345.11	290.43	1821.16	351.09
Formant 3 (Hz)	3928.43	413.19	3857.40	480.39
Formant 4 (Hz)	5488.19	268.56	5483.35	399.01
Formant dispersion (Hz)	1457.53	91.68	1431.33	128.93
Relative amplitude range (dB)	15.22	4.98	21.51	7.71
Relative amplitude change/time (dB/s)	116.52	28.61	180.67	50.57
HNR	2.71	3.05	5.71	2.12
Jitter	0.07	0.03	0.05	0.015
Call duration (s)	0.20	0.05	0.12	0.03
Duration of tonal parts (s)	0.16	0.05	0.11	0.03
Duration of tonal parts (%)	77.85	19.33	90.51	3.10

**Table 2:** Parameters selected for Discriminant function analysis (DFA) and results of DFA for grouped calls.

Category	Number of calls in DFA	Parameters selected for DFA	Wilks' Lambda	Canonical correlation	Eigenvalue
	unselected/selected				
a)					
Social context (*N* = 117)	29/88	Call duration	0.553	Function 1: 0.802	1.807
		Mean fundamental frequency	0.729		
		Relative amplitude changes/time	0.631		
		Formant 2	0.682		
b)					
Sex (*N* = 112)	28/84	Jitter (a)	0.892	Function 1: 0.590	0.533
		HNR	0.736		
		Formant 3	0.874		
		Duration of tonal parts	0.753		
c)					
Males (*N* = 82)	21/61	Maximum fundamental frequency	0.083	Function 1: 0.965	13.383
		Formant dispersion	0.164	Function 2: 0.912	4.925
Females (*N* = 28)	7/21	Mean fundamental frequency	0.054	Function 1: 0.988	39.517
		Formant 2	0.027	Function 2: 0.965	13.484

*N* =  number of calls per category, (a) indicate that parameter was not used to calculate discriminant functions.

### Discrimination of Sexes

Compared to females, greeting croops of males were characterised by significantly lower jitter (Mann-Whitney U: *U* = 210.0; *P*<0.001), but significantly higher values for HNR (*U* = 371.0; *P*<0.001), the third formant (*U* = 376.0; *P*<0.001), and tonality (*U* = 446.0; *P*<0.001). Of all unselected greeting croops, 91.6% were discriminated correctly for sex based on HNR, the third formant, and tonality (Table 2b). With prior probabilities of 74.0% for males and 26.0% for females, the level of classification for sex was significantly different (χ^2^ = 19.5011; df = 1; *P*<0.001). The nested pDFA on the same data set (112 calls of 18 individuals) classified 77.74 of the cross-validated calls correctly (69.41%, *P* = 0.007), using the acoustic parameters HNR, third formant, and tonality.

### Discrimination of Individuals

For greeting croops in male individuals, only 21.7% of the unselected cases were classified to the correct individual (highest prior probability  = 13.9%). Within female individuals, 50.6% of the unselected greeting croops were assigned correctly (highest prior probability  = 38.6%). In males, maximum fundamental frequency and formant dispersion contributed to classification. In females, mean fundamental frequency and the second formant remained in the analysis (Table 2c). For both male and female individuals, the level of classification was statistically not different from prior probabilities (males: χ^2^ = 1.8029; df = 10; *P* = 0.998, females: χ^2^ = 1.9429; df = 3; *P* = 0.584).

### Playback Experiment

**Figure 2:**
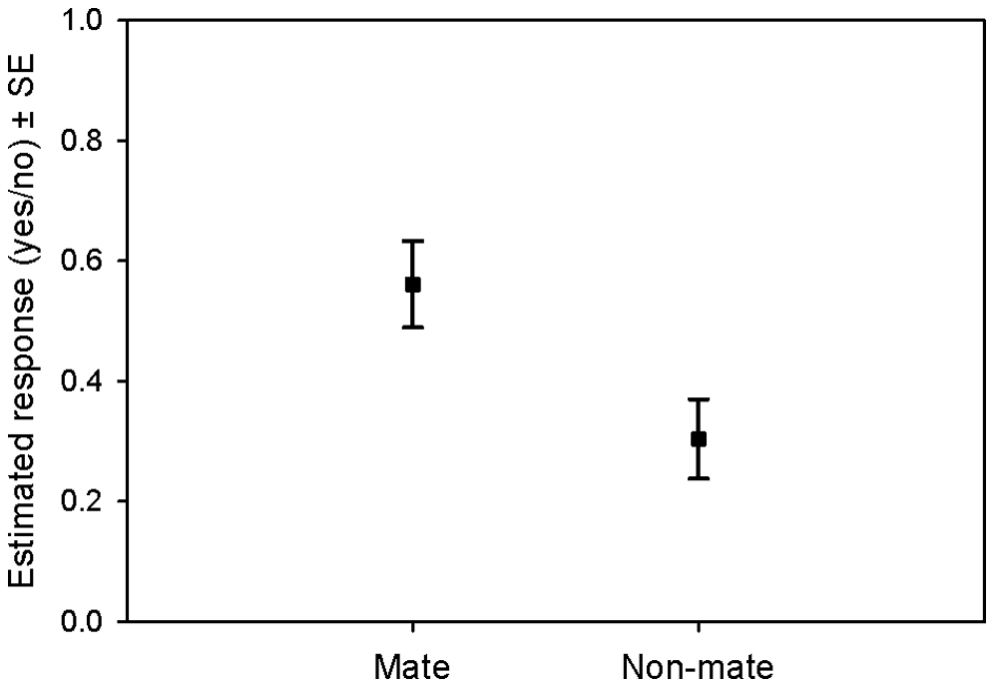
Estimated occurrence of responses ±SE of focal birds (*N* = 12) to the playbacks of mates and non-mates. Values are taken from the GLMM analysis, which controlled for fixed and random effects.

**Figure 3:**
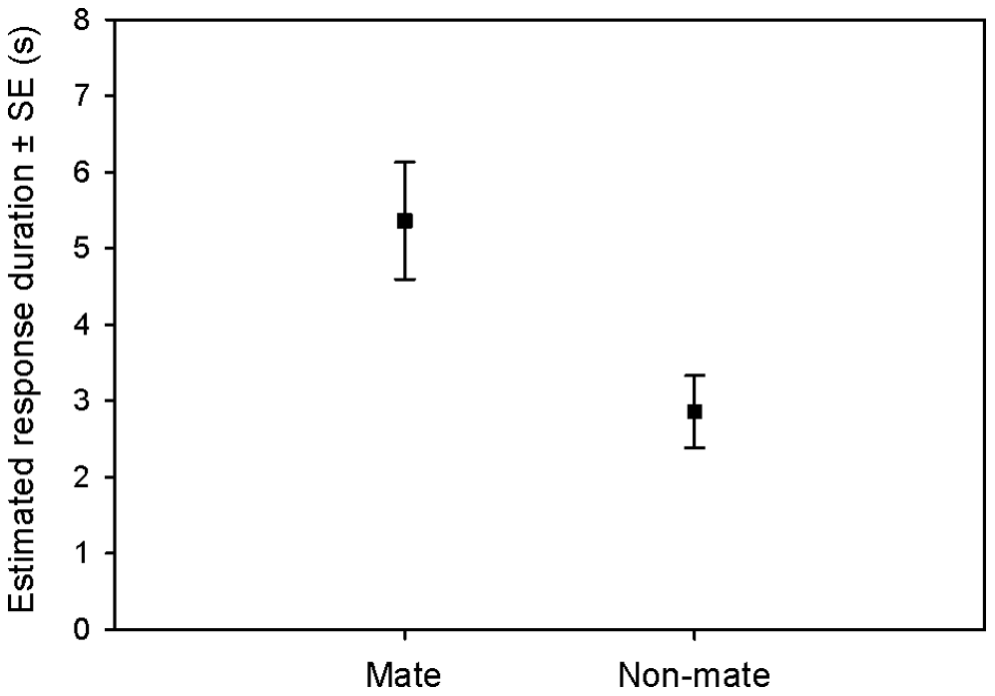
Estimated mean response duration ±SE of focal birds (*N* = 12) to the playbacks of mates and non-mates. Values were retrieved from the GLMM, which controlled for fixed and random effects.

In 55.93% of the trials, focal individuals responded to the playbacks of their mates, whereas a response was shown towards non-mates in 30.51%. The status (mate or non-mate) of the stimulus bird significantly influenced the occurrence of responses (GLMM: df1 = 1, df2 = 116, F = 7.679, *P* = 0.007; Figure 2) and response duration of the focal individual (GLMM: df1 = 1, df2 = 48, F = 14.333, *P*<0.001; Figure 3, Table 3). Focal birds responded significantly more often to their mates by looking up and towards the hidden speaker, and within the cases where responses were shown, significantly longer in response to playbacks of mates than to calls of non-mates.

**Table 3:** AICc-based full and final generalised linear mixed models of response and response duration onto playbacks of mate and non-mate stimuli.

Target	Model	AICc	Coefficients	F	*P≤*
Response (yes/no)	Session + Sequence + Status + Session*Sequence + Session*Status + Sequence*Status (full model)	536.460			
			Intercept	1.696	0.129
			Session	0.020	0.887
			Sequence	0.691	0.407
			Status	0.114	0.737
			Session*Sequence	0.173	0.678
			Session*Status	0.233	0.631
			Sequence*Status	1.089	0.299
	Status (final model)	515.590			
			Intercept	7.679	0.007
			Status	7.679	0.007
Response duration (s)	Session + Sequence + Status + Session*Sequence + Session*Status + Sequence*Status (full model)	116.369			
			Intercept	4.646	0.001
			Session	0.010	0.922
			Sequence	1.166	0.286
			Status	6.670	0.013
			Session*Sequence	0.376	0.543
			Session*Status	0.140	0.710
			Sequence*Status	3.289	0.077
	Status (final model)	97.060			
			Intercept	14.333	0.001
			Status	14.333	0.001

Outcome for all coefficients in the models and their significance are shown (* indicate interactions between factors).

## Discussion

We found that Northern Bald Ibis croop calls differed between social contexts and sexes, but to a lesser extent between individuals. As for the social context, greeting croops were longer, had lower fundamental frequencies, showed higher measures of the second formant, and larger formant dispersion, than agonistic croops. Interestingly, we found that agonistic croops had also higher relative amplitude ranges (intensity measures) and more rapid changes in relative amplitude over time.

In general, arousal influences the acoustic structure of vocalisations due to physiological processes that cause increases in muscles tension related to the control of respiration and vocal organs. This affects mainly temporal and source-related call features, leading to longer calls and higher fundamental frequency with higher arousal (reviewed in [23]). This is in concordance with our results showing higher fundamental frequencies in agonistic croops, which may indicate increased arousal in Northern Bald Ibis during agonistic encounters. Similarly, arousal levels were shown to affect fundamental frequency in the same way for instance in chacma baboon (*Papio cyncephalus ursinus*) vocalisations [25], African elephant rumbles [28], and squirrel monkey (*Saimiri sciureus*) calls [24]. Further support for higher arousal levels in the agonistic compared to the greeting context is provided by wider amplitude ranges and more rapid amplitude changes found in agonistic croops. Increased amplitudes along with higher arousal were also shown for instance in agonistic calls of bison (*Bison bison*) [43] and African elephant rumbles [28]. However, greeting croops were longer than those uttered during agonistic encounters, which contradicts the idea that only physiological arousal makes the difference between the two calls, and is in contrast to previous results [36]. One possible explanation for this could be seasonal variation and concomitant hormonal patterns. Our recordings were conducted in March, during courtship period, whereas Pegoraro and Föger [36] recorded calls over the course of four years, which might have masked seasonal effects. In European Eagle Owls (*Bubo bubo*), duration of call bouts is longest in the courtship phase, before birds lay eggs and start incubation [44]. Grey Partridges (*Perdix perdix*) show a significant increase in call duration towards the breeding period [45], which in males is affected by testosterone [46]. Mated male and female Northern Bald Ibis in the study population were shown to have similar patterns of androgen metabolites with a slight rise at the beginning of the breeding season [47]. High testosterone levels in Buff-branded Rails (*Gallirallus philippensis*) correlate with courtship but not necessarily with agonistic interactions [48]. Therefore, the durations of aggressive calls do not necessarily need to be affected by elevated testosterone levels. This may also apply to agonistic croops of Northern Bald Ibis in our study, leading to the differences found in duration between greeting and agonistic croops.

However, considering that we recorded the calls in March, at the beginning of the breeding season, both agonistic and greeting croops might have been uttered in a state of high arousal. During the start of the breeding season, Northern Bald Ibis engage in courtship and competition over potential mates, and both males and females show elevated corticosteroid metabolites, indicating increased stress levels [47]. Therefore, arousal might be relatively constant and high in both contexts, while emotional valence would differ. As emotional valence is less easily studied than arousal, only few and mostly contradictory studies exist on valence in humans and mammals (for a review see [23]), and evidence for birds is so far lacking.

Although little is known about the biological meaning of formants and their production in birds, formants are used in parrot vocal production (Monk Parakeets, *Myiopsitta monachus*: [49]), and are perceived in conspecific vocalisations (Oilbirds, *Steatornis caripensis*: [6], Whooping Cranes, *Grus americana*: [50]), indicating that formants could be produced and attended to by birds in the same way as in humans and mammals. In agonistic croops, we found lower second formant frequencies than in greeting croops. In chacma baboons grunts, one study is consistent with our findings [25], while another one found lower second formant frequencies with positive valence (infant handling) [51]. To our knowledge, there are no comparable studies in birds that would allow direct comparisons with our measurements. Altogether, we found various structural call differences in Northern Bald Ibis, where two classes of croops are used in two different social contexts, and therefore serve different functions. These acoustic differences may well encode the motivation of the sender, however, physiological parameters are required to confirm these assumptions.

We investigated whether calls could be classified by individual and sex differences in greeting croops. Our results concur with Pegoraro and Föger [36], showing significant differences in sex. These differences were due to filter-related acoustic measures (third formant, HNR, tonality). As Northern Bald Ibis males and females do not differ in body size and weight, source-related acoustic parameters were not expected to differ. However, females have slightly shorter necks and beaks, which in addition to possible differences in body posture might account for the filter-related parameters we found. Classification results were not significantly higher than expected by chance (compared to prior probability values) for male and female individuals. However, when testing pair partners in the playback experiment, birds responded more often and longer to calls of their mates than to calls of other colony members by looking up and towards the speaker. No vocalisations were recorded as behavioural responses, which might be due to the absence of a simultaneous visual stimulus. The fact that focal birds responded to greeting calls of their mates even though calls were recorded two years prior to the playbacks either supports the assumption that greeting croops contain temporarily stable individual differences, which was also shown for Eagle Owls [52], or that calls are remembered for several years irrespective of their changes, as shown in Common Ravens (*Corvus corax*: [53]). Thus, our results indicate that Northern Bald Ibis are capable to recognise their mates by greeting croops.

In conclusion, our results offer insight into the communication system of the critically endangered Northern Bald Ibis, and show that the acoustic structure of croops is mediated by social context, and that these structural variations are, to a large extent, consistent with those found in other studies investigating motivational differences. Further, Northern Bald Ibis seem to have individually distinct stable features in their greeting croops, and are able to recognise their mates via these greeting croops. Croop calls in Northern Bald Ibis provide a promising study case to investigate motivation and emotion in birds. Future studies should focus on the physiological basis underlying agonistic and greeting contexts and its direct effect onto the vocal structure of croop calls.

## Supporting Information

File S1 Detailed description of the sound analysis conducted in Praat.
(DOC)
