# Supplementary material for: Mate Recognition and Expression of Affective State in Croop Calls of Northern Bald Ibis (Geronticus eremita)
Source: PLoS One. 2014 Feb 5;9(2):e88265. doi: 10.1371/journal.pone.0088265 (PMC3914947; doi:10.1371/journal.pone.0088265)
Supplement: File S1 — Detailed description of the sound analysis conducted in Praat. (DOC) [file pone.0088265.s001.doc]

Supporting Information referred to in

Georgine Szipl, Markus Boeckle, Sinja A.B. Werner and Kurt Kotrschal

**Mate Recognition And Expression of Affective State in Croop Calls of Northern Bald Ibis (*Geronticus eremita*)**

**Materials and Methods**

Data Analyses

We applied a stop Hann band filter (0-80 Hz) to reduce background noise created by microphone handling or wind. We then extracted the visual fundamental frequency with the ‘To pitch (cc)’ command (time step = 0.01s, minimum fundamental frequency = 200 Hz, maximum fundamental frequency = 500 Hz). The extracted fundamental frequency contour was visually compared with the spectrogram of the call. In cases where automated fundamental frequency tracking did not match the visual control, measurements were manually adjusted. In a next step, mean, maximum and minimum fundamental frequency were extracted and fundamental frequency range was calculated by subtracting minimum from maximum fundamental frequency.

We measured call duration manually from the oscillogram by extracting the complete call according to the on- and offset of amplitude peaks, as start and end of the call have low signal-to-noise ratios. Additionally, we retrieved the duration and percentage of the tonal parts of the complete call, calculated by dividing duration of tonal part by call duration multiplied by 100. The cumulated fundamental frequency variation during the call was calculated by adding all frequency shifts from the fundamental frequency contour, which then was standardised by calculating frequency shift per second [1].

To calculate formant estimates we selected the call and retrieved the formant measurements with the ‘Extract visible formant contour’ command within the edit window (maximum formant = 6500 Hz, maximum number of formants = 4, window length = 0.015s, pre-emphasis = 50 Hz). Mean values for formants one to four were retrieved. We calculated mean formant dispersal (fd), by measuring the spacing between consecutive formants one to four in the frequency spectrum (fd = ((f2-f1)+(f3-f2)+(f4-f3))/3). To check if automated formant retrieval was correct we compared the spectrogram of the resampled call (sampling frequency = 13 000 Hz, precision = 50 samples) with the linear predictive coding (command ‘To LPC’) by drawing both into the picture view. Peak overlaps were verified in automatically saved EPS files; only calls with overlapping LPC and spectrogram peaks were included in the analyses.

Relative amplitude measurements were extracted with the ‘To Intensity’ command. Amplitude range was calculated by subtracting minimum amplitude from maximum amplitude. Amplitude changes over time were calculated as the sum of all amplitude changes measured divided by call duration.

The harmonics-to-noise ratio (HNR), a relation of energy in harmonics to energy in noise, was measured to quantify the harmonic parts in relation to the noisy parts of the call. HNR was measured using the ‘To Harmonicity (cc)’ command (time step = 0.01, minimum fundamental frequency = 250 Hz, silence threshold = 0.1, periods per window = 1). Low HNR levels represent main energy in harmonic parts.

For measuring random variations of periodicity of the acoustic source, which is reported to correlate with age, we calculated local jitter by first selecting the call file plus the interpolated fundamental frequency contour and then, retrieving a Point Process object with the ‘To PointProcess (cc)’ command. Afterwards, original call, interpolated fundamental frequency and Point Process object were selected to execute the ‘Voice report’ command (fundamental frequency range = 200 to 500 Hz, maximum period factor = 1.3, maximum amplitude factor 1.6, silence threshold = 0.45).

**Reference**

1. Reby D, McComb K (2003) Anatomical constraints generate honesty: Acoustic cues to age and weight in the roars of red deer stags. Anim Behav 65: 519-530.
